# Interactive Application of Data Glove Based on Emotion Recognition and Judgment System

**DOI:** 10.3390/s22176327

**Published:** 2022-08-23

**Authors:** Wenqian Lin, Chao Li, Yunjian Zhang

**Affiliations:** 1 School of Media and Design, Hangzhou Dianzi University, Hangzhou 310018, China; 2 College of Computer Science and Technology, Zhejiang University, Hangzhou 310027, China; 3 College of Control Science and Technology, Zhejiang University, Hangzhou 310027, China

**Keywords:** human-computer interactive, data glove, virtual hand, emotion driven, test

## Abstract

In this paper, the interactive application of data gloves based on emotion recognition and judgment system is investigated. A system of emotion recognition and judgment is established based on the set of optimal features of physiological signals, and then a data glove with multi-channel data transmission based on the recognition of hand posture and emotion is constructed. Finally, the system of virtual hand control and a manipulator driven by emotion is built. Five subjects were selected for the test of the above systems. The test results show that the virtual hand and manipulator can be simultaneously controlled by the data glove. In the case that the subjects do not make any hand gesture change, the system can directly control the gesture of the virtual hand by reading the physiological signal of the subject, at which point the gesture control and emotion control can be carried out at the same time. In the test of the manipulator driven by emotion, only the results driven by two emotional trends achieve the desired purpose.

## 1. Introduction

Though virtual reality (VR) is a way for human beings to interact with computers and complex data, its main purpose is to allow users to enter the virtual environment, wherein they can have the same experience and feeling as in real life. VR involves many fields and advanced technologies.

VR systems can be divided by different aspects. In terms of system functionality, the essential function of a VR system is environment simulation, so it can be applied to many fields such as military, medicine, and so on. At present, there are three kinds of VR systems: (1) systems used for simulation exercise or training in military field, (2) systems for planning and designing places and environment in the field of architecture, and (3) entertainment equipment and high-immersion systems in the entertainment field. In terms of interaction mode and user immersion mode, VR systems can be divided into non-interactive experience, human-virtual environment interactive experience, and group-virtual environment interactive experience. In terms of data input channels, VR can be divided into platform data, model data, perception data, and control data. In terms of interaction mode and interaction equipment, VR can be divided into four types: scene display, force/touch interaction, tracking and positioning, and walking interaction. The scene display type includes a helmet such as the popular VR glasses, desktops, projections, handhelds, and free stereoscopic displays. The force/touch interaction type includes the data glove with transmission functions, joysticks with force feedback, etc. The tracking and positioning type includes source and non-source tracking and positioning systems. The walking interaction type includes pedal walking and ground walking. In the design of VR system, attention should be paid to the elements of multi-perception, immersion, interaction, and imagination space.

Hand gesture recognition is an interactive type of VR system that relies on sensor technologies such as the electromyographic (EMG) and inertial measurement unit (IMU). There have been numerous studies on hand gesture recognition based on EMG and IMU. For example, Kundu et al. [1] presented a hand gesture based control of an omnidirectional wheelchair using IMU and myoelectric units as wearable sensors, and recognized and classified seven common gestures using a shape-based feature extraction and a Dendrogram Support Vector Machine (DSVM) classifier. Classification involved recognizing the activity pattern based on periodic shape of trajectories of the triaxial wrist tilt angle and EMG-RMS from the two selected muscles. Classification accuracy of 94% was achieved by DSVM classifier on ‘k’ fold cross validation data of 5 users. Zhang et al. [2] computed a deep learning technique known as the long short-term memory (LSTM) algorithm to build a model to classify hand gestures by training and testing the collected IMU, EMG, and finger and palm pressure data. The experimental results showed an outstanding performance of the LSTM algorithm. Song et al. [3] proposed a force myography (FMG), EMG, and IMU-based multi-sensor fusion model for hand motion classification, and evaluated the feasibility by motion classification accuracy and qualitative of subjects’ questionnaires. They showed that the offline classification accuracy of adopting combined FMG-EMG-IMU was 81.0% for the 12 motions, which was obviously higher than single sensing modality; that is, only EMG, FMG, and IMU were 69.6, 63.2, and 47.8%, respectively. Jiang et al. [4] presented the design and validation of a real-time gesture recognition wristband based on surface EMG and IMU sensing fusion, which can recognize 8 air gestures and 4 surface gestures with 2 distinct force levels. The results showed that classification accuracies for the initial experiment were 92.6% and 88.8% for air and surface gestures, respectively, and there were no changes in accuracy results during testing 1 h and 1 day later. Yang et al. [5] applied the multivariate variational mode decomposition to extract the spatial-temporal features from the multiple channels to the EMG signals and used the separable, convolutional neural network for modeling by proposing an extensible two-stage machine learning lightweight framework for multi-gesture task recognition. The experimental results for a 52 hand gestures recognition task showed that the average accuracy on each stage is about 90%. Alfaro and Trejos [6] presented a user-independent gesture classification method combing EMG data and IMU data. They obtained average classification accuracies in the range of 67.5–84.6%, with the Adaptive Least-Squares Support Vector Machine model obtaining accuracies as high as 92.9%. Wu et al. [7] proposed a wearable system for recognizing American Sign Language (ASL) by fusing information from an inertial sensor and surface EMG sensors. Four popular classification algorithms were evaluated for 80 commonly used ASL signs on four subjects. The results showed 96.16% and 85.24% average accuracies for intra-subject and intra-subject cross session evaluation, respectively, with the selected feature subset and a support vector machine classifier. Shin et al. [8] studied a myoelectric interface that controls a robotic manipulator via neuromuscular electrical signals generated when humans make hand gestures. They proposed a system that recognizes dynamic hand motions and configuration of a hand over time. The results showed that the average real-time classification accuracy of the myoelectric interface was over 95.6%. Shahzad et al. [9] studied the effects of surface EMG signal variation on the performance of a hand motion classifier due to arm position variation, and explored the effect of static position and dynamic movement strategies for classifier training. A wearable system was made position aware (POS) using IMU for different arm movement gestures. The results showed the effectiveness of the dynamic training approach and sensor fusion techniques to improve the performance of existing stand-alone surface EMG-based prosthetic control systems. Ordóñez Flores et al. [10] proposed a new methodology and showed its particular application to the recognition of five hand gestures based on 8 channels of electromyography using a Myo armband device placed on the forearm. Romero et al. [11] presented the application of hand gestures and arm movements to control a dual rotor testbench. Chico et al. [12] employed a hand gesture recognition system and the inertial measurement unit integrated in the Myo armband sensor as a human-machine interface to control the position and orientation of a virtual six-degree-of-freedom (DoF) UR5 robot.

Hand gesture recognition mainly includes two methods. One is gesture recognition based on data gloves (i.e., the motion characteristics such as the bending degree), angle, and displacement of each key joint of the hand are obtained through the motion sensor and are then inversed to the system database as much as possible. The other is image-based gesture recognition (i.e., the image data of the hand are collected through camera), wherein the background segmentation and motion modeling are carried out through image recognition, and the hand motion is ultimately restored in the computer. The above two methods have their own advantages and disadvantages. Data gloves need subjects to wear external equipment, which may affect the user interaction experience and have delay in data processing, but they have strong anti-interference to data acquisition, more accurate data acquisition, and are not easily affected by the external environment. The image recognition method is more convenient, and the user’s operation is more natural, but it has certain requirements for the environment and is easy to be disturbed by environmental factors. In this paper, the data glove is selected as the interactive device because its data acquisition is more accurate and the sensor used in this paper must contact the user’s hand to obtain the physiological signal. In addition, data gloves are easy to implement modification measures, such as adding additional sensors, and have more advantages and pertinence than other interactive devices in hand movement.

Some achievements have been made in the research and development of data gloves, such as 5DT data gloves, cyberglove force feedback data gloves, measurand high-precision data gloves, X-IST music simulation data gloves, etc. Tarchanidis et al. [13] presented a data glove equipped with a force sensor with a resolution of 0.38 N and a sensitivity of 0.05 V/N. Kamel et al. [14] implement data glove from motion animation to signature verification and showed a high accuracy in finding the similarities between genuine samples as well as those differentiated between genuine-forgery trials. Yoon et al. [15] presented a data glove with adaptive mixture-of-experts model and showed the excellent performance and adaptability through tests. Kim et al. [16] used a data glove to present a sign language recognition system and indicated that the system was useful when employed to smartphones in some situations. Chen et al. [17] presented a data glove with highly stretchable conductive fiber strain sensor, which could recognize various gestures by detecting the finger motion. Fang [18] proposed a data glove to recognize and capture the gestures of 3-D arm motion, and the test results verified its effectiveness. Lin et al. [19] presented a data glove with characteristics of low cost, high reliability, and easy wearability. Wang et al. [20] presented a data glove with the feedback force control of a safe, lightweight, yet powerful and stable passive force feedback. Li [21] developed a data glove to monitor the hand posture and operated the division between sensor and base signal to decrease the test error induced by instability of light sources. Wu et al. [22] presented a data glove for catching finger joint angles and tested its effectiveness. Sarwat et al. [23] used a data glove to construct an automated assessment system for in-home rehabilitation, helping poststroke patients with a high level of recovery. Takigawa et al. [24] developed a controlled functional electrical stimulation to realize multiple grasping postures with data glove.

The previous research on data gloves has mostly focused on improving the accuracy of motion recognition and pressure simulation of force feedback. However, the study on the data glove which can capture the user’s behavior and obtain the user’s emotion through physiological signal sensor is rare. Therefore, in this paper, a kind of data glove with functions of emotion recognition and interaction between human and computer, or a human and hardware device according to the user’s emotion, is presented. The data glove can be used in medicine, health, military training, academic research, and other fields.

## 2. Classification of Emotion Trends

**Figure 1:**
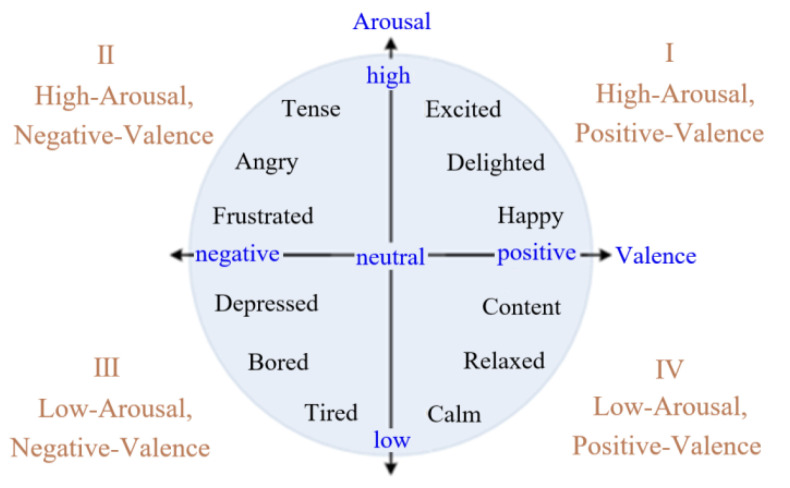
Valence-arousal model.

In order to obtain user’s emotion through physiological signal sensor, a system of emotion recognition is needed, while emotion recognition is based on the emotion evaluation [25]. Here the valence-arousal (V-A) model is used for the emotion classification. In the V-A model, as shown in Figure 1, V and A indicate the degree of emotional pleasure and emotional arousal, respectively. Four poles of the emotion classification model are extracted and used to represent tired, tense, happy, and depressed, respectively. The emotion classification system based on the V-A model is extended to a plane, and four quadrants of the plane stand for high-arousal and positive-valence (quadrant Ⅰ: HAPV), high-arousal and negative-valence (quadrant Ⅱ: HANV), low-arousal and negative-valence (quadrant Ⅲ: LANV), and low-arousal and positive-valence (quadrant Ⅳ: LAPV), respectively.

## 3. System of Emotion Recognition and Judgment

### 3.1. Data Analysis of Physiological Signal (PS)

In the present study, skin electricity and pulse wave are taken as PS. The former is easily disturbed by other signals, so the noise interference should be removed before advancing. In order to facilitate computer analysis and processing, the discrete wavelet transform is used to decompose the signal into different frequency bands through low-pass and high-pass filtering. The unit of the frequency used for the filter is Hertz. The wdencmp function in MATLAB 9.0 R2016a is used to denoise the skin electrical signal, and all segments of skin electrical signal were normalized within the range of 0 to 100.

**Figure 2:**
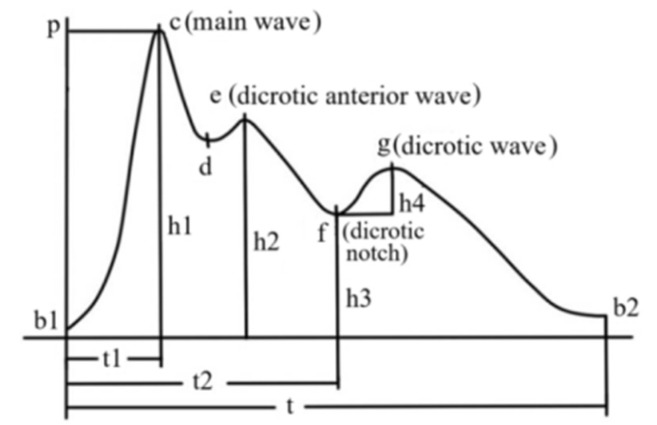
Key feature points of pulse wave.

As shown in Figure 2, the signal of pulse wave is composed of main wave, dicrotic anterior wave, dicrotic notch and dicrotic wave. In the figure, the key feature points include: (1) c (peak systolic pressure), (2) e (starting point of left ventricular diastole), (3) g (maximum pressure point of anti tide wave), (4) d (point of aortic dilation depressurization), (5) f (origin of anti tide wave), and (6) b1 (point of aortic valve opening). The key amplitude includes: (1) main wave h1, (2) dicrotic anterior wave h2, (3) dicrotic notch h3, and (4) dicrotic wave h4. The key time includes: (1) the time from the starting point of waveform period to the peak c point of main wave t1, (2) the time from the starting point of waveform cycle to the lowest point of dicrotic notch t2, and (3) duration of one waveform period t. The pulse wave is smoothed and filtered using Butterworth low-pass filter and the relevant parameters of pulse wave are normalized after filtering.

### 3.2. Extraction of Optimal Feature of PS

Features of PS are divided into a time domain, a frequency domain, and a feature related with physiological processes [26]. The direct fusion of original signal features will result in too much computation. As such, the dimensionality reduction of original signal feature is performed using the method of principal component analysis (PCA) to make the classifier more efficient and accurate in emotion recognition. Principal components are obtained using PCA, and then the weight threshold of each feature of PS on the principal component is taken as the criterion for selecting feature. Finally, some original features that play a major role can be determined as optimal feature subset. After obtaining optimal feature subset, the Pearson correlation coefficient (PCC) is used to judge the relationship between the emotional interval and these features. The PCC is calculated for features of four emotion trends and can be used to draw the significance P of the features. Based on P and correlation coefficient, the normalized threshold of optimal features correlated with emotional trends is determined. These optimal features include BpNN50 (percentage of main pulse wave interval >50 ms), the “range” of skin electrical signal (the mean value of first order difference for skin electrical signal), and 1dmean (mean value of first order difference of skin electrical signal).

### 3.3. Establishment of the System of Emotion Judgment

**Figure 3:**
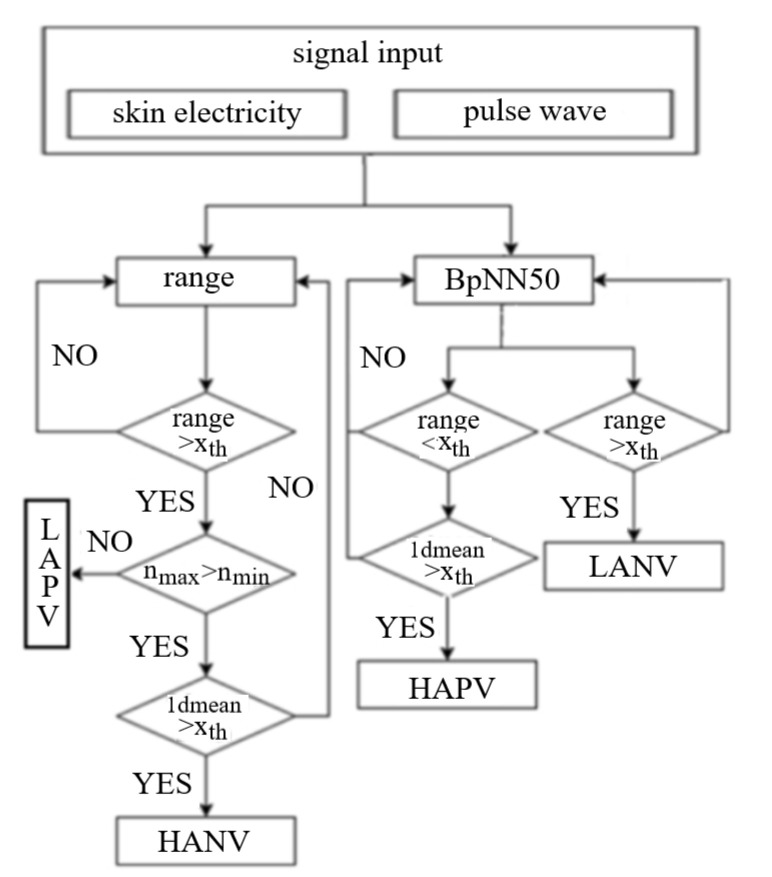
Process of emotion judgment model.

The range of skin electrical signal has a high positive correlation between the two completely opposite emotional trends (i.e., HVLA and LVHA). As such, the skin electrical waveform corresponding to the emotional trend is studied. The results show that it is necessary to add a directional judgment to the range of skin electrical signal. Based on the set of optimal signal feature from the Pearson correlation coefficient, the system of emotion recognition and judgment can be built according to the process as shown in Figure 3.

## 4. Design and Connection of Data Glove

**Figure 4:**
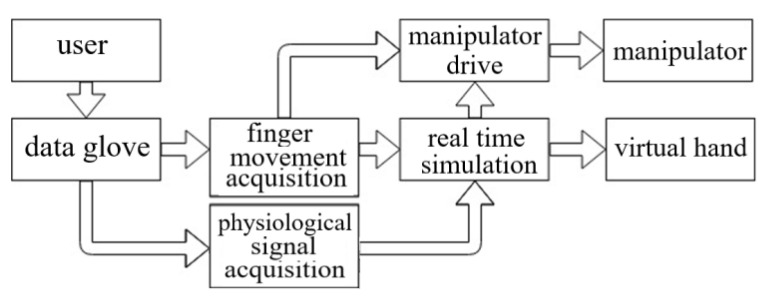
Design framework of data glove with emotion recognition function.

**Figure 5:**
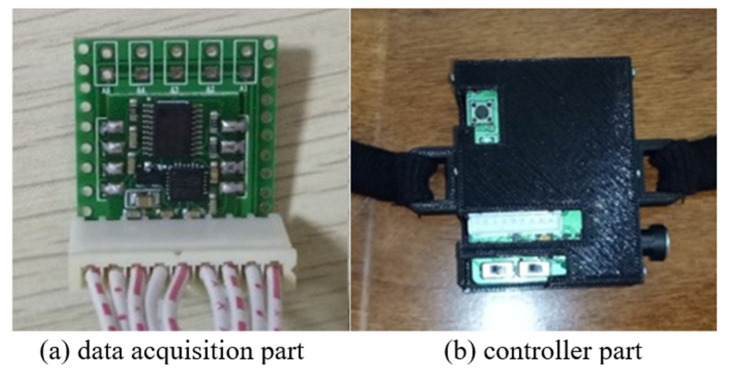
DN-1 composition of data glove module.

**Figure 6:**
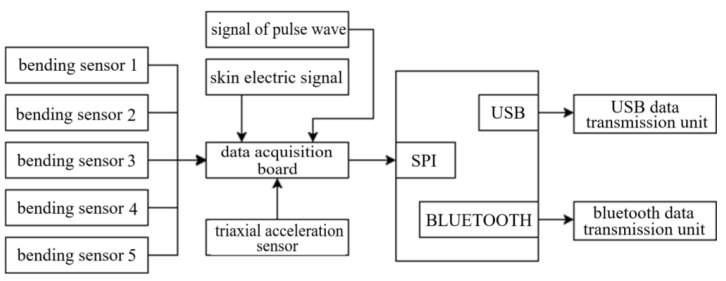
Data glove hardware structure framework.

The design framework of data glove with emotion recognition function is shown in Figure 4 where the data glove equipment consists of data acquisition and the controller. The data are acquired from the finger movement and physiological signal. The data glove customized based on DN-01 data module is taken as an example as shown in Figure 5, wherein the data module is an attitude acquisition board, which is used to collect the information of hand motion such as finger motion parameters, angular velocity of hand rotation, hand rotation acceleration, and angle change. The controller processes and integrates the collected information of hand motion, and then packages the processed data and sends it to the host computer for processing through Bluetooth or USB to serial port. The interface of the attitude acquisition board is connected with the sensor, and the acquisition board and the controller are connected by a flat cable as shown in Figure 6.

**Figure 7:**
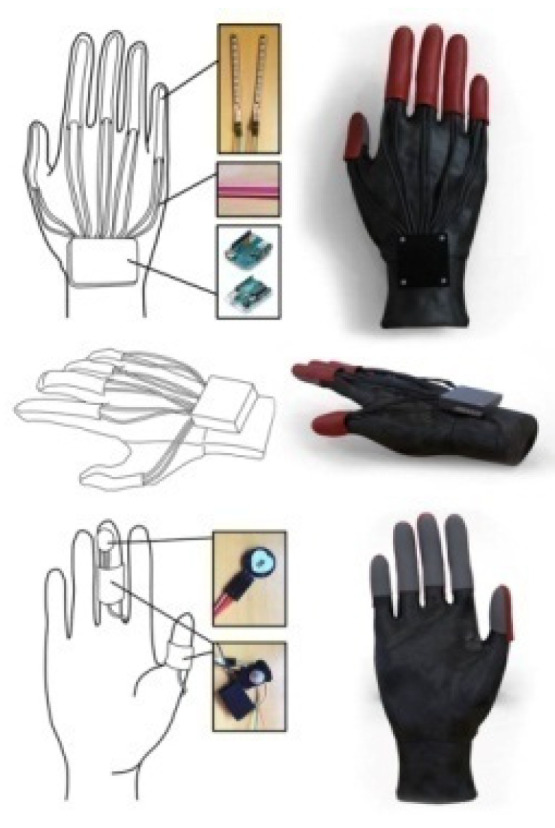
Prototype design of data glove based on emotion recognition of physiological signal.

The data acquisition module in the data glove mainly collects two kinds of data: one is gesture data, and the other is physiological signal data. The prototype design of the data glove is shown in Figure 7, where the sensor of gesture data is Flex2.2 bending sensor which can capture the bending degree of five fingers and the motion posture of the palm, including acceleration, angular velocity, and angle. The length of the bending sensor is 7.7 cm, the non-bending resistance is 9000 Ω, the 90-degree bending resistance is 14,000 Ω, and the 180-degree bending resistance is 22,000 Ω.

**Figure 8:**
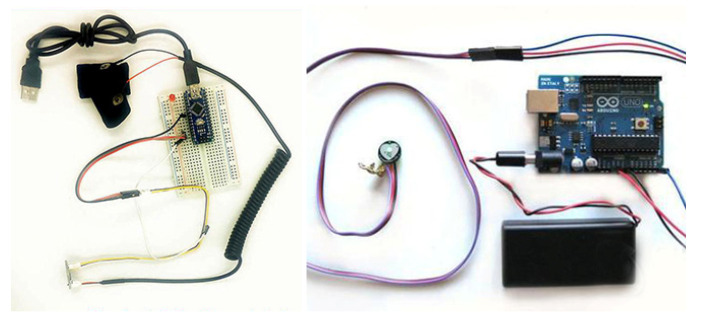
GSR skin electrical kit (**left**) and pulse sensor (**right**).

The skin electrical signal is acquired using a Grove-GSR skin electrical kit, as shown in Figure 8 (left). Two finger sleeves containing electrodes were put on the middle part of the middle finger and the thumb of the left hand, and the frequency of signal sampling was 20 Hz. A pulse sensor, as shown in Figure 8 (right), was used to acquire the signals of the pulse wave and heart rate. The pulse sensor was fixed on the tip of the middle finger of the left hand with a bandage, and the frequency of signal sampling was 100 Hz.

Gestures are reflected by the bending of fingers, and the output format of finger bending is: 0xaa a1 a2 a3 a4 a5 0xbb, where 0xaa and 0xbb are the head and tail of the frame, and a1, a2, a3, a4, and a5 represent the bending data of five fingers from thumb to little thumb, respectively. The data x read on the interface (a1–a5) is the quantization of the voltage value on the bending sensor:

(1) $$x=\frac{V_{x}\times 3.3}{4096}$$

where *V_x_* is voltage at sensor. Based on

(2) $$V_{x}=3.3\times \frac{R}{\left(R+20\right)}$$

the resistance value *R* can be obtained. The value of *R* is proportional to the bending degree of the bending sensor—i.e., the values of a1, a2, a3, a4, and a5 are inversely proportional to the degree of finger bending.

The finger bending data can be analyzed through the following functions: (1) the resume function is used to determine whether the data related to the finger part is received correctly (i.e., the correctness of frame header and tail), (2) finger_calculate function is used to calculate the bending data of the finger, (3) judge function is used to determine whether the finger is bent within a reasonable range (i.e., filtering out wrong motion information), and (4) calculate function is used to process data related to finger bending including bending data of a single finger, storing and recording the data, calculating the offset of bending data, etc.

The acquisition sensors of physiological signal are the skin electrical sensor Grov-GSR skin electric kit (fixed on the middle part of the middle finger and the thumb of the glove) and the pulse sensor (fixed on the tip of the middle finger of the glove). The sensors are connected to the data acquisition module with a wire, and then the acquisition module is connected to the PC end and external hardware equipment through the Bluetooth interface. Unity3D receives the data information transmitted by the data acquisition board through the IO interface.

## 5. Test of Virtual Gesture Change Driven by Emotion

### 5.1. System Design

**Figure 9:**
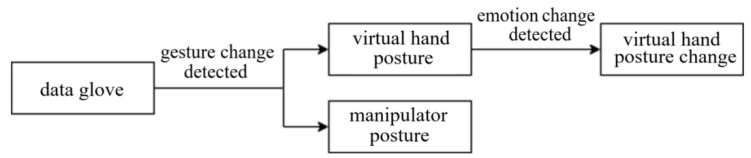
The technological process of the system that virtual gesture change driven by emotion.

The data glove is used to control the hand gesture of the virtual hand and manipulator, and then the gesture of the virtual hand is changed through the awakened emotion of the subjects and compared with the gesture of the manipulator. The technological process of the system is shown in Figure 9, where DN-1 data glove is adopted and two kinds of sensors are added to DN-1 data glove.

**Figure 10:**
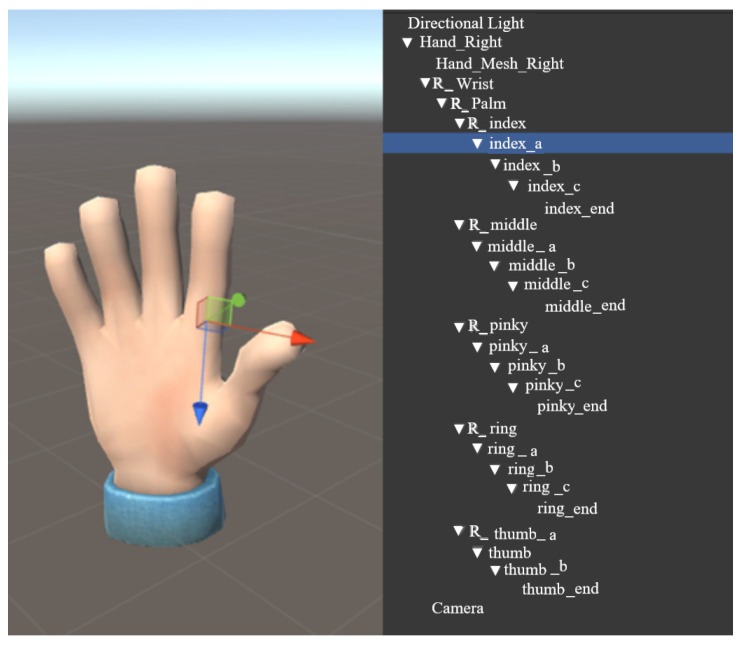
Virtual hand model.

The virtual hand model comes from network shared resources. The fingers of index finger, middle finger, ring finger, and little thumb have 5 movable joint points, respectively, and the thumb has four movable joint points. There are 24 movable joint points for changing the gesture of the hand, as shown in Figure 10.

Each joint of the hand is taken as a changeable unit, and two sets of attitude change systems are loaded for the virtual hand model in Unity3D. One is the gesture system which is related to the data of finger gesture transmitted from the data glove; that is, the virtual hand changes the gesture according to the related data of finger gesture, which is consistent with the subject’s hand gesture. Another set of action templates is the gesture animation file designed in advance; that is, the corresponding gesture action of virtual hand is activated after the activation conditions are met. 

### 5.2. Virtual Hand Control Driven by Emotion

**Figure 11:**
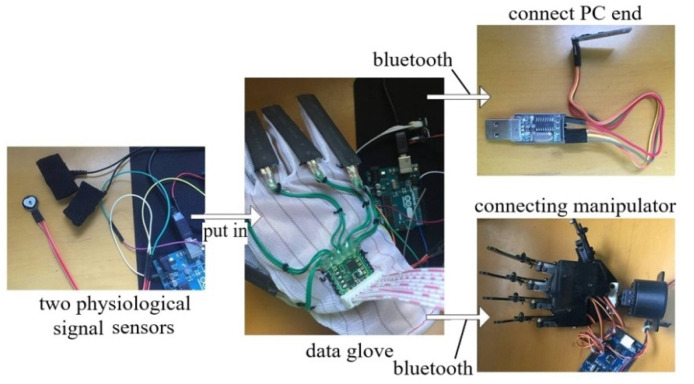
Connection of test equipment that virtual gesture change driven by emotion.

Five subjects, aged between 24 and 30, participated in the test. Music materials were used to awaken the subjects’ emotions in the test. The DN-1 customizable five fingers mechanical claw with the most basic functions is used as external hardware equipment, and the connection of test equipment is shown in Figure 11.

**Figure 12:**
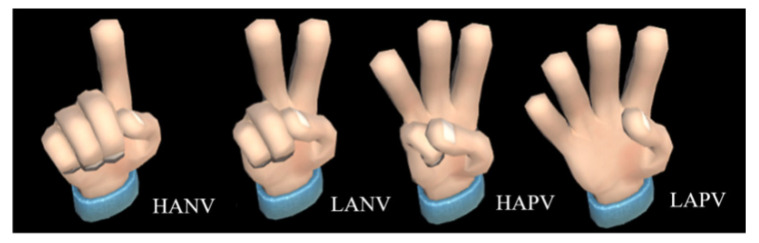
Four hand animation gestures.

The principle and test process are as follows: (1) playing the music with style of terror, sadness, grandeur, and freshness to awake subjects’ emotion; (2) the physiological signals (skin electricity and pulse wave) caused by the subjects’ emotion are collected by sensors placed in the data glove; (3) four emotional trends HANV, LANV, HAPV, and LAPV corresponding to terror, sadness, grandeur, and freshness are detected using the system of emotion recognition and judgment as described in Section 3 based on the physiological signals; (4) the emotion changes of the subjects are detected by the system which is built based on the relationship between the emotion and gesture of the virtual hand; and (5) the system drives the virtual hand to make four animation gestures of “1”, “2”, “3”, and “4” corresponding to HANV, LANV, HAPV, and LAPV as shown in Figure 12. 

**Figure 13:**
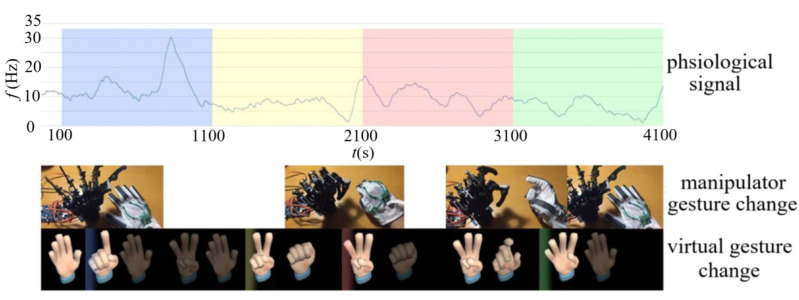
Gesture changes of subject 3 driven by emotion.

The virtual gesture changes of subject 3 as driven by emotion are shown in Figure 13, where we can see the corresponding relationship between physiological signal and virtual gesture change.

A gesture data acquisition module is also placed in the data glove as described in Section 4. The system can drive the virtual hand and the manipulator to make the gesture consistent with the gesture of the subject. In the process described above, when the virtual hand makes the gestures of “1”, “2”, “3”, and “4”, the subjects also make the same gesture, which drives the manipulator also to make the same gesture as shown in Figure 13. Therefore, there is a time deviation between the gestures of the virtual hand and the manipulator as show in Table 1. 

In Table 1, the time deviation between virtual hand change driven by emotion and manipulator change is basically less than 20%, showing that the virtual hand and manipulator can be controlled synchronously through the data glove. When the user’s data glove does not make any gesture change, the system can directly control the gesture of the virtual hand by reading the physiological signal of the subject, and gesture control and emotion control can be carried out at the same time to achieve the desired purpose.

### 5.3. Manipulator Control Driven by Emotion

**Figure 14:**
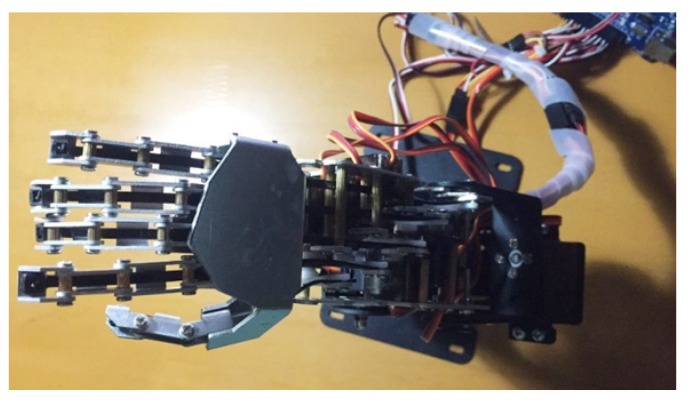
Manipulator in the test.

The manipulator with six degrees of freedom, weight of 4.5 kg, and load capacity of 5 kg is directly controlled using data glove as shown in Figure 14, where the DN-1 data glove is adopted and two kinds of sensors are added to DN-1 data glove.

The control of manipulator is divided into gesture control of finger part and arm part. The control of the finger part can be seen in Section 4, and the arm part controls the movement angle, including the elbow joint (float anglere), the wrist joint (float anglere 1) and the finger root joint on the palm (float anglere 2).

The output angle related data includes acceleration, angular velocity, and angle.

(1) Acceleration:

0 × 55 0 × 51 AxL AxH AyL AyH AzL AzH TL TH SUM(3)

where 0 × 55 and 0 × 51 are the head and tail of the frame; AxL, AyL, and AzL are the low byte of *x*, *y,* and *z* axes; AxH, AyH, and AzH are the high byte of *x*, *y,* and *z* axes; TL and TH are the total data transmission; and SUM is the acceleration output checksum:

0 × 55 + 0 × 51 + AxH + AxL + AyH + AyL + AzH + AzL + TH + TL(4)

where the symbols are the same as those in Equation (3).

(2) Angular velocity:

0 × 55 0 × 52 wxL wxH wyL wyH wzL wzH TL TH SUM(5)

where 0 × 55 and 0 × 51 are the head and tail of the frame; wxL, wyL, and wzL are the low byte of *x*, *y,* and *z* axes; wxH, wyH, and wzH are the high byte of *x*, *y,* and *z* axes; TL and TH are the total data transmission; and SUM is the acceleration output checksum:

0 × 55 + 0 × 52+wxH+wxL+wyH+wyL+wzH+wzL+TH+TL(6)

where the symbols are the same as those in Equation (5).

(3) Angle:

0 × 55 0 × 53 RollL RollH PitchL PitchH YawL YawH TL TH SUM(7)

where 0 × 55 and 0 × 53 are the head and tail of the frame, RollL and RollH are roll angle for *x* axis, PitchL and PitchH are pitch angle for *y* axis, YawL and YawH are yaw angle for *z* axis, TL and TH are total data transmission, and SUM is the acceleration output checksum:

0 × 55 + 0 × 53 + RollH + RollL + PitchH + PitchL + YawH + YawL + TH + TL(8)

where the symbols are the same as those in Equation (7).

The angle related data is parsed by the following function:

(1) port_noanglesure function is used to reverse judgment. The data packet is not the angle packet of the hand.
(2) angle_resume function is used to verify whether the data received by the angle package is correct, and to obtain 11-bit data of angle packet (angle_data0, angle_data1, angle_data2, …, angle_data9, angle_data10).
(3) angledata_calculodegree function is used to convert the received angle data into two-bit angle data in degrees.

**Figure 15:**
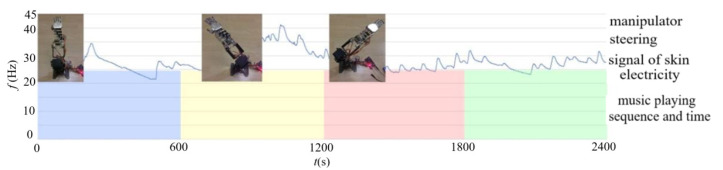
Manipulator steering change driven by emotion.

The angle drive data are replaced with physiological signal data. The driving conditions of the manipulator steering are determined by the system of emotion recognition and judgment as shown in Section 3. The steering settings are up (HANV), left (LANV), right (HAPV), and down (LAPV), respectively. Each piece of music lasts 30 s ± 1 s. The manipulator steering change driven by emotion is shown in Figure 15. We can see that the manipulator completes the steering action only driven by the emotions of LANV and HAPV, and there was no response under the emotion conditions of HANV and LAPV, showing that, although the scheme of manipulator driven by emotion is feasible, it needs to be further improved in the recognition rate of emotion and the response speed of the manipulator.

## 6. Conclusions

In this paper, the interactive application of data glove based on emotion recognition and judgment system is studied. A data glove with multi-channel data transmission based on hand gesture recognition and emotion recognition is constructed. The system of virtual hand control and manipulator driven by emotion is established using Unity3D as a construction tool of computer system. In the test of virtual hand control driven by emotion, the data glove is used to simultaneously control the virtual hand on the PC side and external mechanical claw, while the system of emotion recognition and judgment is only used in the virtual hand control. In the test of the manipulator driven by emotion, the data glove is used to directly control the manipulator, and the arm angle control is replaced by the optimal features of physiological signal. The test results show that the virtual hand and manipulator can be simultaneously controlled by the data glove. The main innovation lies in the discovery that, in the case that the subjects do not make any hand gesture change, the system can directly control the gesture of the virtual hand by reading the physiological signal of the subject, and the gesture control and emotion control can be carried out at the same time. In the test of the manipulator driven by emotion, only the results driven by two emotional trends achieve the desired purpose. Although the system of the manipulator driven by emotion is feasible, it needs to be improved.

## Figures and Tables

**Table 1:** The time deviation between virtual hand change driven by emotion and manipulator change.

Subjects	1	2	3	4
1	−8.44%	+13.42%	−7.12%	−5.03%
2	−14.62%	X	+3.94%	−21.01%
3	−16.38%	−18.93%	+11.27%	−15.50%
4	−15.11%	X	−9.21%	+10.48%
5	−15.57%	−17.81%	−7.44%	x

## Data Availability

Data sharing not applicable.
